# Characterization of La/Fe/TiO_2_ and Its Photocatalytic Performance in Ammonia Nitrogen Wastewater

**DOI:** 10.3390/ijerph121114626

**Published:** 2015-11-17

**Authors:** Xianping Luo, Chunfei Chen, Jing Yang, Junyu Wang, Qun Yan, Huquan Shi, Chunying Wang

**Affiliations:** 1 School of Resources and Environmental Engineering, Jiangxi University of Science and Technology, Ganzhou 341000, China; E-Mails: ccfjxnc@126.com (C.C.); 18270733906@163.com (J.Y.); Wangjunyu1026@163.com (J.W.); yanqun8219893@163.com (Q.Y.); 13177767724@163.com (H.S.); cywang@jxust.edu.cn (C.W.); 2 Western Mining Co., Ltd., Xining 81006, China; 3 Jiangxi Key Laboratory of Mining & Metallurgy Environmental Pollution Control, Jiangxi University of Science and Technology, Ganzhou 341000, China; 4 Faculty of Engineering, University of Alberta, Edmonton, AB T6G2V4, Canada

**Keywords:** characterization, photocatalysis, La/Fe/TiO_2_, ammonia nitrogen wastewater

## Abstract

La/Fe/TiO_2_ composite photocatalysts were synthesized by Sol-Gel method and well characterized by powder X-ray diffraction (XRD), scanning electron microscopy (SEM), nitrogen-physical adsorption, and UV-Vis diffuse reflectance spectra (UV-Vis DRS). It is interesting that the doped catalysts were in anatase phase while the pure TiO_2_ was in rutile phase. In addition, the composites possessed better physical chemical properties in photocatalytic activity than pure TiO_2_: stronger visible-light-response ability, larger specific surface area, and more regular shape in morphology. The photodegradation results of ammonia nitrogen indicate that: the La/Fe/TiO_2_ had higher catalytic activity to ammonia nitrogen waste water compared pure TiO_2_ and the other single metal-doped TiO_2_. pH 10 and 2 mmol/L H_2_O_2_ were all beneficial to the removal of ammonia nitrogen by La/Fe/TiO_2_. However, the common inorganic ions of Cl^−^, NO_3_^−^, SO_4_^2−^, HCO_3_^−^/CO_3_^2^^−^, Na^+^, K^+^, Ca^2+^ and Mg^2+^ in water all inhibited the degradation of ammonia nitrogen. By balance calculation, at least 20% of ammonia nitrogen was converted to N_2_ during the 64.6% removal efficiency of ammonia nitrogen.

## 1. Introduction

In recent years, a large amount of ammonia nitrogen wastewater was discharged into the water with the mining of rare earth metals in Gannan area, China. Excessive amounts of ammonia nitrogen in water would cause many harmful effects, and the treatment of ammonia nitrogen wastewater is a concern [1]. The methods of chemical precipitation, blow-off, and adsorption are commonly used for the treatment of ammonia nitrogen wastewater at low concentrations. Chemical precipitation method intends to reduce the water solubility of ammonia nitrogen by the formation of indissoluble salt; blow-off method is typically used NaOH to adjust pH to basic of wastewater and ammonia nitrogen would exist in the form of free ammonia (NH_3_). Then, ammonia nitrogen would escape from aqueous solution to the atmosphere, which might be difficult to recover and cause the secondary pollution in atmospheric. Adsorption method is mainly based on the ion exchange of NH_4_^+^ with other cationic ions, which is a reversible process but the exchange capacity is limited [2,3,4]. The ideal treatment result is that ammonia nitrogen would totally convert to nitrogen. Marco [5] reported that partial of ammonia nitrogen was oxidized to nitrogen by photocatalytic oxidation technology using TiO_2_ as the catalyst.

As one of the most promising technologies of treating pollutants in water, photocatalytic oxidation process has received intense attention in many fields and has been researched widely on the environmental protection, health care, building materials and other industries, especially on the photodegradation of pollutants [6,7,8,9,10,11,12]. Compared with other advanced oxidation technologies such as Fenton oxidation and ozone oxidation, catalytic wet oxidation, and electrochemical oxidation, photocatalytic oxidation technology is non-toxic and has good stability [13]. Undoubtedly, among the various semiconductor photocatalytic materials, titanium dioxide (TiO_2_) is the typical photocatalyst due to its good chemical and biological stability, low cost, ease of availability, and significantly photocatalytic activity under ultraviolet irradiation [14,15,16,17]. However, its application remains limited because of its high electron-hole recombination rate in photocatalytic process; another shortcoming of TiO_2_ is that it only absorbs ultraviolet light no longer than 387.5 nm, which only accounts for about 4% of sunlight [18,19,20]. To resolve these problems, one important way is to extend the photoresponse of TiO_2_ into visible regions, which has already been studied [11,21,22]; and another important way is to adulterate some amount of metal or nonmetal elements into TiO_2_ to increase the migration efficiency of photogenerated electrons and decrease the recombination rate of electron-hole pairs. There are several reports of the photodegradation of ammonia nitrogen by modified TiO_2_ [12,23,24,25,26]. TiO_2_ doped with Fe could utilize visible light wavelengths and effectively produced hydrogen from the decomposition of aqueous NH_3_ while TiO_2_ doping with rare earth ions playing the key role in the ammonia photocatalytic decomposition [23,24].

Combined with the advantage of iron and rare earth doped TiO_2_, La-Fe-codoped TiO_2_ was chosen to prepare and the main objectives of this paper are in four aspects: firstly, to investigate the physics chemical properties of La-Fe-codoped TiO_2_ prepared by Sol-Gel method and characterized by XRD, SEM, EDS, and UV-Vis DRS; secondly, to study the photocatalytic activity of prepared doped TiO_2_ to ammonia nitrogen; thirdly, to discuss the effect of reaction solution pH, H_2_O_2_, and common inorganic ions on the degradation of ammonia nitrogen; finally, to disclose the conversion products of ammonia nitrogen during the photodegradation process.

## 2. Materials and Methods

### 2.1. Reagents

Tetrabutyl titanate (Ti(OC_4_H_9_)_4_), lanthanum nitrate (La(NO_3_)_3_), ferric nitrate (Fe(NO_3_)_3_) and ammonium chloride (NH_4_Cl) were of analytical grade and purchased from National Medicine Group Chemical Reagent Co., Ltd., Shanghai, China. Anhydrous ethanol (CH_3_CH_2_OH) was purchased from Shanghai Zhan Yun Chemical Co., Ltd., Shang hai, China.

### 2.2. Modification Methods

The photocatalysts were prepared by Sol-Gel method. The mixture solution of 8.5 mL tetrabutyl titanate dissolved in 20 mL anhydrous ethanol with stirring for 30 min was noted as solution A. Another solution containing 20 mL ethanol, 1.5 mL deionized water, and metal salts (La(NO_3_)_3_ and/or Fe(NO_3_)_3_) in the required stoichiometry was noted as solution B. Solution B was pumped into solution A by half drop (*ca.* 0.05 mL) per second at *ca.* 30 °C. The mixture was hydrolyzed at room temperature for a period of time under vigorous stirring and finally the translucent sol was formed. The gel was prepared by aging the sol for two days at room temperature. The dry gel was gained after drying at 80 °C for 2 h. Finally, the gel was calcined at 500 °C at the heating rate of 2.5 °C/min in the muffle furnace for 2 h and was ground into powders for use.

### 2.3. Characterization

Powder X-ray diffraction (XRD) data were recorded on a D/Max-3c X-ray diffraction meter at 40 kV and 40 mA for monochromatized Cu *K*α (λ = 1.5418 Å) radiation. Scanning electron microscopy (SEM) measurements were carried out on S-4800 type of field emission scanning electron microscope with energy dispersive spectrometer (EDS). The BET surface areas of the samples were obtained from the automatic analyzer (JW-004A, Beijing JWGB Sci.&Tech. Co.,Ltd, Beijing, China). UV-Vis diffuse reflectance spectra were achieved using a UV-Vis spectrophotometer (UV-2550, Shimadzu China Co., Ltd., Japan), and the absorption spectra were referenced to BaSO_4_.

### 2.4. Photocatalytic Removal Experiments and Analytical Methods

The photocatalytic degradation experiments were carried out in a XPA-7 photochemical reactor (Xujiang Electrical Mechanical Plant, Nanjing, China). The irradiation was provided by a 500 W Mercury lamp (Institute of Electric Light Source, Beijing, China), which mainly radiated 365 nm wavelenght of light and was positioned in the cylindrical quartz cold trap. The system was cooled by circulating water and maintained at room temperature. Before the irradiation, the suspension was magnetically stirred for 30 min in the dark to ensure adsorption equilibrium of ammonia nitrogen on the catalysts. For all the reactions, the irradiation lasted for 300 min. Approximately 5 mL of reaction solution was taken at given time intervals and centrifuged. The supernatant was analyzed by Nessler’s reagent spectrophotometry [27] and the removal efficiency (*R*) was calculated by Formula (1) as follows:

(1) $$R=(C_{0}-C)/C_{0}\times 100\%$$

where *C*_0_ is the initial concentration of ammonia nitrogen and *C* is the concentration at reaction time *t* (min). In order to study the conversion of ammonia nitrogen, NO_2_-N and NO_3_-N were also detected by spectrophotometry methods [28,29]. In addition, the effects of H_2_O_2_ and common ions (Cl^−^, HCO_3_^−^/CO_3_^2^^−^, NO_3_^−^, SO_4_^2−^, Na^+^, K^+^, Ca^2+^, Mg^2+^) in natural waters on ammonia nitrogen removal were investigated. Besides, all the experiments were performed at least twice and the mean values were reported.

## 3. Results and Discussion

### 3.1. Characterization

#### 3.1.1. XRD

**Figure 1:**
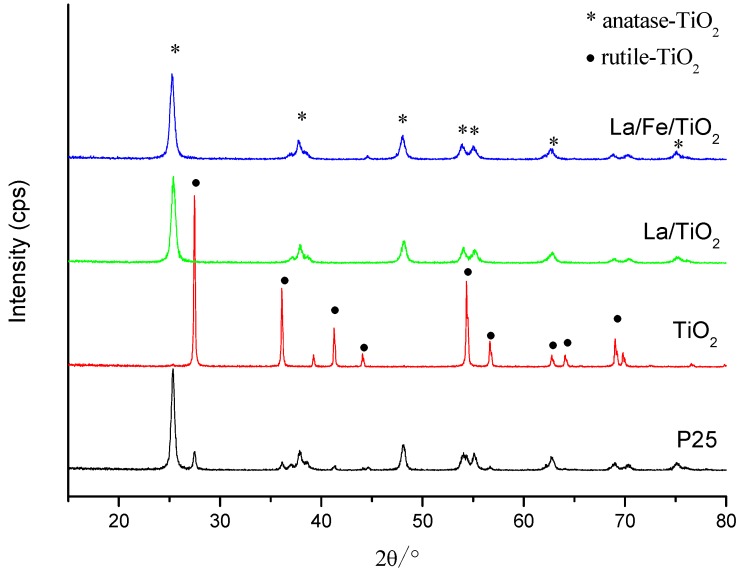
XRD patterns of P25, TiO_2_, La/TiO_2_ and La/Fe/TiO_2_.

The XRD patterns of P25, pure TiO_2_, La/TiO_2_ and La/Fe/TiO_2_ composites are shown in Figure 1. The major peaks at 2θ values of 25.3°, 37.9°, 48.0°, 53.8°, 54.9°, and 62.5° corresponded to diffractions of the (101), (004), (200), (105), (211), and (204) planes of anatase TiO_2_ while the major peaks at 27.5°, 36.1°, 39.2°, 41.3°, 44.1°, 54.4°, 56.7°, 62.8°, 64.1°, 65.6°, 69.1° corresponded to diffractions of the (110), (101), (111), (210), (211), (220), (002), (310), and (112) planes of rutile TiO_2_. This showed that the pure TiO_2_ prepared existed in the rutile phase while the doped catalysts in the anatase phase whether La/TiO_2_ or La/Fe/TiO_2_. It is interesting to find that the doped rare earth Lanthanum changed the crystal structure of TiO_2_ from rutile to anatase. Compared to P25, the crystallinity of doped TiO_2_ decreased as shown in Figure 1. Besides, there are no peaks for the formation of composite metal oxides such as La_2_O_3_ or Fe_2_O_3_ in doped TiO_2_, which might be ascribed to the fact that the concentration of La-doping and/or Fe-doping was so low and the overlapping of diffraction peaks due to TiO_2_, La_2_O_3,_ and/or Fe_2_O_3_.

#### 3.1.2. UV-Vis DRS

**Figure 2:**
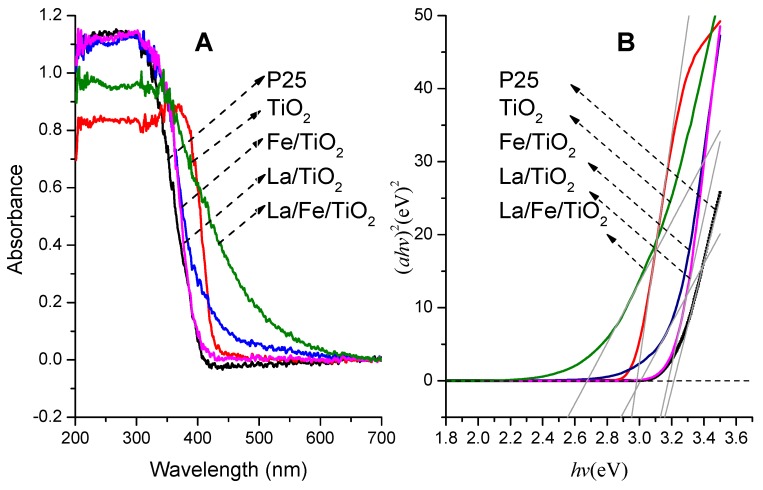
UV-Vis DRS of P25, TiO_2_, La/TiO_2_ Fe/TiO_2_, and La/Fe/TiO_2_ ((**A**) Absorbance; (**B**) Plots of (α*h*υ)^2^ *versus* (*h*υ) for catalysts).

The UV-Vis DRS spectra of P25, TiO_2_, La/TiO_2_, Fe/TiO_2,_ and La/Fe/TiO_2_ are depicted in Figure 2A. All the doped powders showed a redshift compared to P25 while undoped TiO_2_ exhibits an absorption edge to the visible light region due to the rutile phase. Besides, there is an obvious change of light absorption of La/Fe/TiO_2_ from ultraviolet to visible light due to the La-Fe-codoping. The redshift phenomenon indicates that the modified TiO_2_ broaden the scope of light response as anatase phase. In other words, the visible-light-response catalysts overcome the disadvantage of the broadband gap to a certain extent [30]. In addition, the absorption data were analyzed using the following well-known formula for near-edge optical absorption of semiconductors [31].

α = A (*h*υ − *E*_g_)^n^/*h*υ (2)

where α is the absorption coefficient, (*h*υ) is the photon energy, A is a constant, *E*_g_ is the optical gap, and the value of n is 1/2 for TiO_2_ [32]. To estimate the optical band gap, the plot of (α*h*υ)^2^ *versus* (*h*υ) is shown in Figure 2B. The *E*_g_ values are 3.22, 2.98, 3.01, 3.18, and 2.68 eV for P25, TiO_2_, La/TiO_2_, Fe/TiO_2_ and La/Fe/TiO_2_, which indicates that the doped material did broaden the scope of light response of compared with pure TiO_2_ and P25.

#### 3.1.3. Surface Morphology Analysis

**Figure 3:**
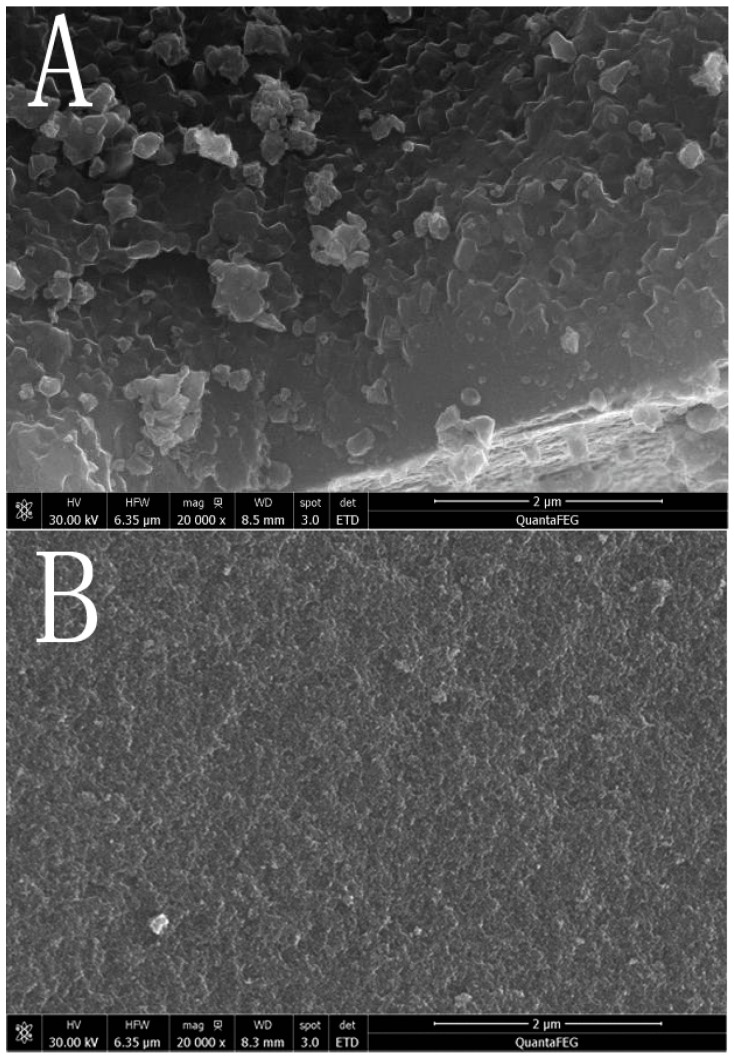
SEM images of TiO_2_ (**A**) and La/Fe/TiO_2_ (**B**).

As seen from Figure 3, pure TiO_2_ (Figure 3A) exhibits irregular shape and is agglomerated badly. However, after La and Fe co-doped, the reunion phenomenon is abated and is a relatively flat surface. Compared to Figure 3A and 3B, the more serious reunion phenomenon of pure TiO_2_ might be based on the large amounts of hydroxyl groups on the surface of pure TiO_2_ which would result in the strong hydrogen bonding between particles [33].

Further data for the composition of La/Fe/TiO_2_ photocatalysts were obtained by EDS: La accounted for 3.62% while Fe accounted for 0.62%. The result is consistent with the UV-Vis DRS and proves that elements lanthanum and iron were all loaded on the surface of TiO_2_.

#### 3.1.4. Specific Surface Area Analysis

The specific surface area of P25, TiO_2_, La/TiO_2_, and La/Fe/TiO_2_ is 48.12, 65.57, 78.36, and 120.74 m^2^/g respectively. A significant increase in specific surface area of the doped samples was observed. The increase in specific surface area after doping may be caused by the decrease in the crystallite size of TiO_2_, as described in the XRD and SEM part, which is in agreement with Anandan’s report that doping of rare earth could increase the surface area of TiO_2_ [34].

### 3.2. Degradation Performance of Ammonia Nitrogen Wastewater

**Figure 4:**
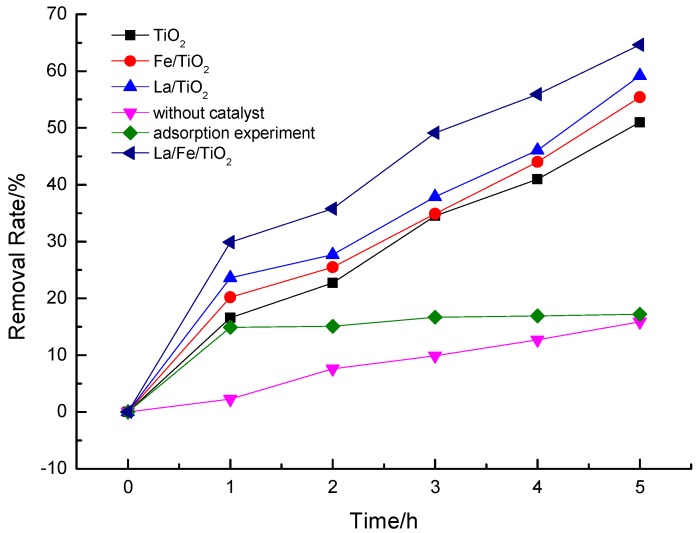
The curves of photocatalysis of NH_4_^+^-N at different conditions.

A series of control experiments were designed to investigate the photocatlytic activity of prepared doped composites, and all the experiments were carried out with the same conditions of pH (*ca.* 10), catalyst amount (1 g/L), and 500 W mercury lamp. Figure 4 shows the result. After 5 h, about 15% of ammonia nitrogen was removed by direct photolysis or escaping from the reaction solution by magnetic stirring, while above 50% was removed by photocalytic degradation with catalyst. Furthermore, the doped catalysts showed higher photocatalytic activity on ammonia nitrogen removal than pure TiO_2_ by the analysis of first-order reaction kinetics as shown in Table 1. The best removal efficiency of ammonia nitrogen reached 64.6% with La/Fe/TiO_2_.

**Table 1:** Reaction kinetics constant of catalysts.

Catalyst	*k*	*R*^2^
TiO_2_	0.137	0.990
Fe/TiO_2_	0.150	0.988
La/TiO_2_	0.163	0.976
La/Fe/TiO_2_	0.196	0.984

Anandan [34] reported that small particle size, high surface area, high surface roughness, and porous surface of La-doped TiO_2_ and the suppression of electron-hole recombination by La^3+^ were the reasons for the high photocatalytic activity of La-doped TiO_2_, the characterization result of this study is consistent with their conclusion. Besides, Fe^3+^ is born of the electron capture trap [23]. Under the synergy of La^3+^ and Fe^3+^, the electron-hole pairs produced from catalyst under irradiation could be effectively separated and the catalytic activity of La/Fe/TiO_2_ was improved.

#### 3.2.1. Effect of Different pH

**Figure 5:**
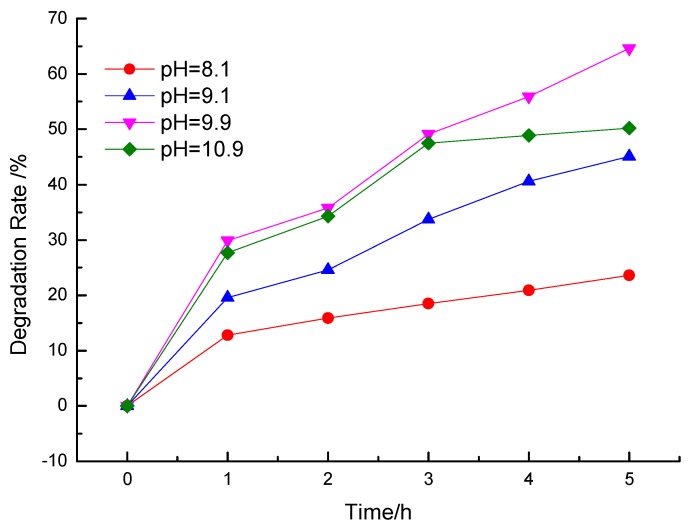
The effect of pH on the degradation of NH_4_^+^-N with La/Fe/TiO_2_.

The initial pH of the reaction solution might influence the surface charge of La/Fe/TiO_2_ and the existing form of ammonia nitrogen in water and finally affect the ammonia nitrogen removal efficiency. Firstly, the number of OH^−^ increases with the pH increases gradually, and more ·OH would be generated induced by La/Fe/TiO_2_, resulting in promoting the removal rate of ammonia nitrogen. Secondly, there are two forms of ammonia nitrogen in water: NH_3_·H_2_O and NH^4+^. Proportion of NH_3_·H_2_O molecules increases as the pH increases in the solution. Thirdly, the space steric hindrance of NH_3_ is smaller than that of NH_4_^+^, which is more conducive to the reaction of NH_3_ with ·OH. At last, the pH_PZC_ (point of zero charge) of La/Fe/TiO_2_ is about 6.4 by analysis of Zeta potential. So, it is difficult for the attraction of ammonia molecules onto the surface of the catalyst in an acidic condition. All the analysis above demonstrates that ammonia nitrogen was removed rapidly in alkaline environment [35]. However, as shown in Figure 5, it did not favor the catalytic reaction at pH 10.9, which might be due to that excessive OH^−^ in the solution. The following experiments would be performed at pH *ca.* 10.

#### 3.2.2. Effect of H_2_O_2_

**Figure 6:**
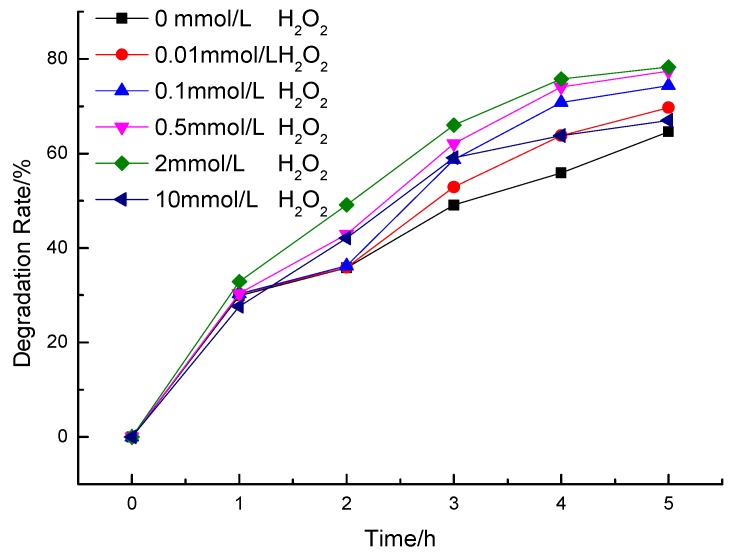
The effect of H_2_O_2_ on the degradation of NH_4_^+^-N with La/Fe/TiO_2_.

H_2_O_2_ is usually applied as a stimulator in TiO_2_ photocatalysis system to enhance the rate of photocatalytic oxidation [36,37]. In order to investigate the effect of H_2_O_2_ addition on ammonia nitrogen degradation by La/Fe/TiO_2_, experiments were conducted by varying the initial H_2_O_2_ concentration in the range of 0.01 to 10 mmol/L (0.01, 0.1, 0.5, 2, 10 mmol/L). As shown in Figure 6, addition of H_2_O_2_ promoted the removal rate of ammonia nitrogen. The removal rate reached 78.3% with H_2_O_2_ of 2 mmol/L. It is well known that H_2_O_2_ has strong absorbance in the range of 200–350 nm and could produce ·OH under UV irradiation (Formula (2)). Besides, as a kind of strong oxidizer, H_2_O_2_ can effectively capture the photoproduction electrons of TiO_2_ conduction belt and be converted to ·OH as Formula (3). So, the degradation was accelerated with the addition of H_2_O_2_.

(2) $${\text{H}}_{2}{\text{O}}_{2}\overset{h\upsilon }{\to }2\cdot \text{OH}$$

(3) $${\text{H}}_{2}{\text{O}}_{2}+e^{-}\to \cdot \text{OH}+{\text{HO}}^{-}$$

However, excessive H_2_O_2_ would exhaust the generated ·OH in the reaction solution (Formulas (4) and (5)) [38] to reduce the promotion.

(4) $${\text{H}}_{2}{\text{O}}_{2}+\cdot \text{OH}\to {\text{H}}_{2}\text{O}+\cdot {\text{HO}}_{2}$$

(5) $$\cdot {\text{HO}}_{2}+\cdot \text{OH}\to {\text{H}}_{2}\text{O}+{\text{O}}_{2}$$

#### 3.2.3. Effects of Inorganic Ions

**Figure 7:**
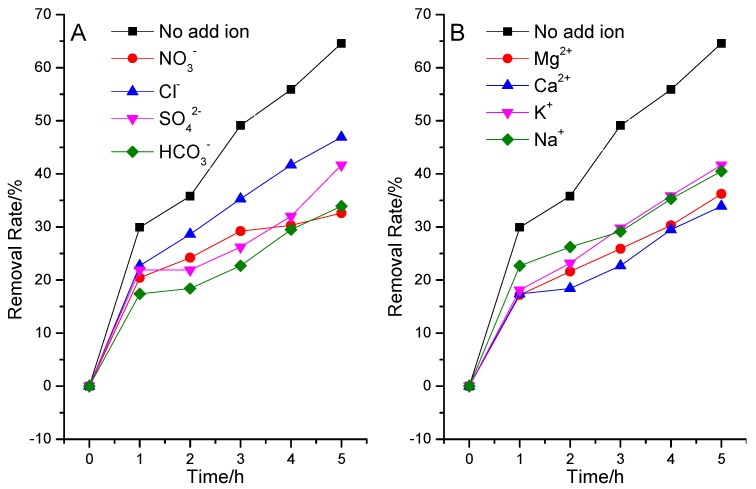
The effect of ions on the degradation of NH^4+^-N with La/Fe/TiO_2_.

There are eight common inorganic ions in natural water [39], including Na^+^, K^+^, Ca^2+^, Mg^2+^, Cl^−^, SO_4_^2−^, NO_3_^−^, and HCO_3_^−^/CO_3_^2^^−^, and the concentrations are all 0.1 mmol/L. They might affect the removal of pollutants in water. Results of the effects of cations and anions on ammonia nitrogen degradation are shown in Figure 7.

As seen from Figure 7, at the same experimental conditions, all kinds of inorganic ions showed an obvious inhibitory effect on ammonia nitrogen removal. Sörensen indicated that NO_3_^−^ acted as an “inner filter” and reduces the UV light intensity in the photoreactor [40]. Thereby, addition of NO_3_^−^ decreased the degradation rate of pollutant in the reaction system. Besides, the inhibited effect increased as the reaction went on. The reason might be that excessive NO_3_^−^ was produced from the conversion of ammonia nitrogen (the content will be discussed below). It was reported that HCO_3_^−^/CO_3_^2^^−^ is an effective ·OH scavenger [40]. It can react with ·OH to produce carbonate radicals, which are weak oxidizing reagents that hardly react with other pollutant molecules. Therefore, HCO_3_^−^/CO_3_^2^^−^ displayed distinct inhibition effect on the degradation of ammonia nitrogen by La/Fe/TiO_2_. Cl^−^ and SO_4_^2−^ also could react with ·OH like HCO_3_^−^/CO_3_^2^^−^, but the reaction ability was lower than NO_3_^−^, and the inhibited effect was smaller than HCO_3_^−^/CO_3_^2^^−^. Since SO_4_^2−^ is double charged, it may display higher inhibition ability than Cl^−^.

Na^+^, K^+^, Ca^2+^, and Mg^2+^ are all in the highest and stable oxidation state and cannot capture electrons or holes in solution. It is hypothesized that these metal ions would not show significant impacts on the photo degradation of ammonia nitrogen by La/Fe/TiO_2_. As shown in Figure 7B, the four metal ions displayed inhibition effects on ammonia nitrogen removal, which could be attributed to the effect of Cl^−^ ions co-present in the solution. The metal ions were used in their chloride salts. As described above, Cl^−^ ions might inhibit the photo degradation due to the reaction with ·OH. Since K^+^ and Na^+^ are in the same elemental main group, they have similar properties. KCl showed similar effect as NaCl. In addition, Ca^2+^ and Mg^2+^ also have similar properties and they (CaCl_2_ and MgCl_2_) displayed similar trends. Furthermore, Ca^2+^ and Mg^2+^ displayed higher inhibition effects than NaCl and KCl at the same mole concentrations. This is expected since the concentration of Cl^−^ in CaCl_2_ and MgCl_2_ solutions was twice of that in NaCl and KCl solutions [41]. Besides, Mg^2+^ and Ca^2+^ tend to form precipitation and adhere to the surface of the catalyst to reduce the photocatalytic efficiency in weak alkaline conditions [42].

#### 3.2.4. Analysis of Degradation Products of Ammonia Nitrogen

WuJie [43] mentioned that the process of photodegradation of inorganic nitrogen in water would generate highly reactive ·OH and O_2_^−^ and other reactive oxygen species, which have photocatalytic ability. Inorganic nitrogen ions can induce a series of REDOX reactions, mainly containing NH_4_^+^ oxidation and NO_3_^−^ reduction (Formulas (6–10)). However, the specific mechanism is still not yet confirmed, and it needs further research.

(6) $${\text{NH}}_{4}^{+}+2{\text{OH}}^{-}\to {\text{NO}}_{2}^{-}+3{\text{H}}_{2}$$

(7) $$2{\text{NO}}_{2}^{-}+{\text{O}}_{2}\to 2{\text{NO}}_{3}^{-}$$

(8) $${\text{NO}}_{3}^{-}+2{\text{H}}^{+}+2e_{ab}^{-}\to 2{\text{ON}}_{2}^{-}+{\text{H}}_{2}\text{O}$$

(9) $${\text{NO}}_{3}^{-}+10{\text{H}}^{+}+8e_{ab}^{-}\to {\text{NH}}_{4}^{+}+3{\text{H}}_{2}\text{O}$$

(10) $$2{\text{NO}}_{3}^{-}+12{\text{H}}^{+}+10e_{ab}^{-}\to 2{\text{N}}_{2}+3{\text{H}}_{2}\text{O}$$

**Figure 8:**
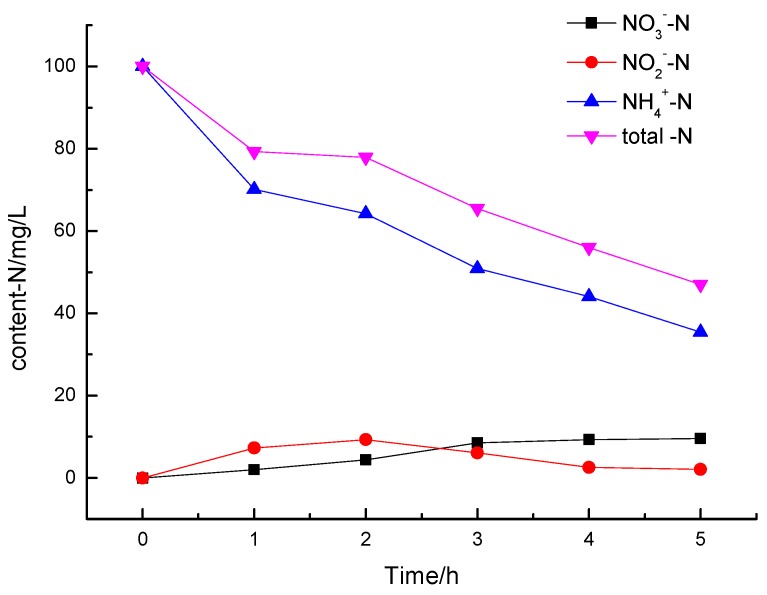
The conversion curves of the NH_4_^+^-N during the photodegradation process.

NO_3_-N and NO_2_-N were detected as the photocatalytic degradation products of ammonia nitrogen wastewater as the following Figure 8. After 300 min of photocatalytic reaction using La/Fe/TiO_2_ as the photocatalyst, the concentration of ammonia nitrogen reached to 34.96 mg/L by the initial concentration of 100.67 mg/L and the conversion rate was 64.6%. During the degradation process, 9.56 mg/L of NO_3_-N and 2.07 mg/L of NO_2_-N were generated. Considering the escape free ammonia and adsorption part onto the catalyst’s surface, it is proposed that at least 20% of ammonia nitrogen was converted to N_2_ according to the mass balance of the total nitrogen [44].

## 4. Conclusions

La-Fe-codoped catalyst demonstrates better physical chemical properties in photocatalytic activity than pure TiO_2_: first of all, the doped catalysts were in anatase phase while the pure TiO_2_ was in rutile phase; second, the composites possessed strong visible-light-response ability; third, La/Fe/TiO_2_ had larger specific surface area and more regular shape in morphology. Furthermore, the doped catalysts indicated higher photocatalytic degradation ability to ammonia nitrogen wastewater: the removal rate of ammonia nitrogen reached to 78.3% at the conditions of pH 9.9, 100.67 mg/L of ammonia nitrogen, 1 g/L of catalyst, and 2 mmol/L of H_2_O_2_. Besides, the common inorganic ions in water all inhibited the degradation of ammonia nitrogen. At last, it is proposed that there at least 20% of ammonia nitrogen was converted to nitrogen gas during the photodegradation process with 64.6% removal efficiency of ammonia nitrogen.
